# The Distributional Characteristics of M2 Macrophages in the Placental Chorionic Villi are Altered Among the Term Pregnant Women With Uncontrolled Type 2 Diabetes Mellitus

**DOI:** 10.3389/fimmu.2022.837391

**Published:** 2022-03-21

**Authors:** Muqiu Zhang, Dong Cui, Huixia Yang

**Affiliations:** Department of Obstetrics and Gynaecology, Peking University First Hospital, Beijing, China

**Keywords:** placenta, macrophage, type 2 dabetes mellitus, chorionic villi, decidua

## Abstract

**Aim:**

No definite conclusions have been drawn regarding how prolonged exposure to hyperglycemia affects the distribution of macrophages in the placenta, especially in pregnant women with uncontrolled type 2 diabetes mellitus (T2DM). Herein, we explored the distributional characteristics of placental M2 macrophages, including hofbauer cells (HBCs) in the chorionic villi and decidual macrophages, in pregnant women with uncontrolled T2DM.

**Methods:**

Six healthy singleton pregnancies and five uncontrolled T2DM singleton pregnancies were collected. Multicolor immunofluorescence and immunohistochemistry were performed to record M1 macrophages by CD80 and CD86, the general M2 macrophages by CD163, M2a macrophages by CD163 and DG-SIGN, M2b macrophages by CD163 and CD86, and M2c macrophages by CD163 and CD206. Meanwhile, the monocyte marker of CD14 and the general macrophage marker of CD68 were also documented on placenta.

**Results:**

In the chorionic villi and decidua, the most common infiltrated macrophages was the general M2. There were only few M1 and M2b macrophages distributed in the placenta of both the healthy and uncontrolled T2DM groups. The infiltrated degree of M2c macrophages was moderate in chorionic villi and decidua. The uncontrolled T2DM and healthy pregnant women had a comparable amount of M2c macrophages infiltration in the chorionic villi (p = 0.158). Notedly, in both of the healthy and uncontrolled T2DM pregnant women, the predominant subtype of M2 macrophages in the chorionic villi was M2a, where it mainly infiltrated around vessels and syncytiotrophoblasts. The uncontrolled T2DM pregnant women had more M2a macrophage infiltration than the healthy pregnant women (p = 0.016). The M2a macrophages in the decidua of the uncontrolled T2DM group were similar to those of the normal group (p = 0.800). Meanwhile, it was in the chorionic villi but not the decidua, that the CD68^+^ macrophages and CD14^+^ M2a macrophages were also elevated in the uncontrolled T2DM group (p = 0.035 and 0.044, respectively).

**Conclusion:**

These results confirmed that the M2 macrophages exhibited increased in the chorionic villi of pregnant women with uncontrolled T2DM. The subsets of M2 macrophages in the placental decidua were similar between uncontrolled T2DM pregnant women and normal groups. It may provide a basis for exploring the functions of different subsets of macrophages in the placental chorionic villi.

## Introduction

Hyperglycemia in pregnancy (HIP) is the most common metabolic disturbance among pregnant women (1). In 2021, it was estimated that one in every six live births worldwide was affected by HIP (2). Only 16% of HIP is the result of pregestational diabetes mellitus (PGDM), which is defined as the existence of type 1 or type 2 diabetes mellitus (3). The remaining 84% of HIP is attributed to gestational diabetes mellitus (GDM) (3). The duration and degree of hyperglycemic exposure and the timing of the onset of exposure are important factors to monitor in the course of pregnancy. Early exposure during fetal organogenesis and placental development has more severe and lasting consequences than later exposure, such as birth defects and perinatal morbidity (4, 5). Perinatal morbidity may ultimately be due to placental immaturity and extensive villous immaturity coupled with compromised function when exposed to a hyperglycemic environment during early pregnancy (6–8). The placenta is an important mediator between hyperglycemic exposure and maternal and fetal complications.

Macrophages are the second most abundant immune cells in the human placenta (9). Physiologically, placental macrophages constantly keep polarization and play a key role in the regulation of pregnancy and fetal development (9). In the placenta, macrophages accumulate mainly in the chorionic villi and decidua. Macrophages of the chorionic villi of the feto-placental unit are also known as hofbauer cells (HBCs) (10–12). HBCs are detected in the villi of the placenta as early as 4 weeks post-conception (13). During the early pregnancy, HBCs originate from mesenchymal progenitor cells in the stroma or monocyte progenitor cells in the hypoblast-derived yolk sac (14, 15). In the second and third trimesters, HBCs differentiate from circulating monocytes in the fetus (16, 17). The HBCs are polarized into M2-like macrophages, namely, M2a, M2b, and M2c polarity subtypes, which play an important role in placental vasculogenesis and angiogenesis (18). M2a macrophages possess tissue repair and immunoregulating features, while M2b macrophages support humoral immunity and allergic reactions (19). M2c phenotype remodel the extracellular matrix and suppress immunity to induce anti-inflammatory reactions (19). By contrast, decidual macrophages derive from monocyte progenitors differentiated from hematopoietic pluripotent stem cells. Decidual macrophages migrate from bone marrow to the bloodstream of maternal side of the placenta, where they further maturate (13). Decidual macrophages represent a skewed phenotype of M2 macrophages producing IL-10 throughout pregnancy (20, 21). HBCs and decidual macrophages, both of which are placental macrophages, are completely different in terms of their origins and functions.

Recent studies have claimed that the disturbed glucose gradient and insulin resistance associated with GDM might alter the normal phenotype distribution of macrophages in the placenta (22). Compared with those in a normal group of the term pregnant women, placental macrophages in pregnant women with GDM consist mainly of the M2a and M2b phenotypes (22). However, it has been reported that the proportion of M2a phenotype HBCs is reduced after onset of uncontrolled type 1 diabetes mellitus (T1DM) during pregnancy (23, 24). On the other hand, macrophages in the decidua are natural candidates for contributing to tissue remodeling at the maternal–fetal interface (9). Their prominent roles as pathogen sensors and immune effector cells suggest a central role in the inflammatory response to pathological stress on the placenta (9). As one category of PGDM, pregnant women with type 2 diabetes mellitus (T2DM) are exposed to hyperglycemia at an early gestational age and in combination with serious complications. Several studies emphasizing the complexity of macrophages distribution and biology in the placenta of pregnant women with GDM and T1DM have come to different conclusions. Few studies have been conducted to explore the distributional characteristics and function of placental macrophages in pregnant women with T2DM, especially when hyperglycemia is out of control.

In the present study, multicolor immunofluorescence was performed to detect the expression conditions and compare the distributional characteristics of macrophages in chorionic villi and decidua between the term pregnant women with uncontrolled T2DM and the normal group. We found that M2a macrophages and CD14^+^ M2a macrophages in the chorionic villi of pregnant women with uncontrolled T2DM were increased. However, the subsets of macrophages in the placental decidua were similar between uncontrolled T2DM patients and normal groups.

## Materials and Methods

### The Inclusion Process

The Peking University First Hospital review board approved this study (2013[752]). Informed consent was obtained from all the participants. The present study included 6 healthy singleton pregnancies and 5 uncontrolled T2DM singleton pregnancies (**Table 1**). The 5 T2DM pregnant women were treated with short-acting insulin before three meals and long-acting insulin at bedtime. In addition, one of them took metformin combined with insulin. All of the above pregnant women received prenatal care and delivery at the Peking University First Hospital. The diagnostic criteria of T2DM were as follows: (1) the presence of one or more symptoms, namely, excessive thirst, polyuria, unexplained weight loss, hunger plus a fasting plasma glucose level >7.8 mmol/L or a random plasma glucose level >11.1 mmol/L; (2) at least two elevated plasma glucose concentrations on occasions, namely, a fasting level of >7.8 mmol/L, a random level of >11.1 mmol/L and a concentration of >11.1 mmol/L after 2 h of oral glucose tolerance testing; and (3) regular therapy with hypoglycemic drugs (subcutaneous insulin and/or oral hypoglycemic drugs) (25). The outcomes of glycosylated albumin and glycosylated hemoglobin are shown in **Table 2**. The glycosylated hemoglobin (%) and glycated albumin (%) levels of the uncontrolled T2DM patients were 6.6 ± 0.9/16.2 ± 2.8, 6.2 ± 0.3/16.3 ± 0.9 and 7.0 ± 0.9/18.4 ± 5.9 in the first, second and third trimesters, respectively. We excluded pregnant women with a history of severe pregnancy complications, namely, hypertension, preeclampsia, eclampsia, cardiovascular illness, symptomatic infectious diseases, chronic disorders, and fetal malformation.

**Table 1:** Information of pregnant women.

	Normal (n = 6)	Uncontrolled T2DM (n = 5)	p-value
Pre-pregnancy body mass index (kg/m^2^)	23.5 ± 1.6	26.7 ± 3.0	0.057
Third trimester body mass index (kg/m^2^)	27.8 ± 1.1	30.0 ± 1.7	0.135
Gestational age (weeks ± days)	39 ± 1	38 ± 5	0.936
Placenta weight (g)	680 ± 152.8	622 ± 90.4	0.622
Birth weight (g)	3,388 ± 427	3598 ± 564	0.750
Fetal sex	M4, F2	M4, F1	

**Table 2:** The outcomes of glycosylated albumin and glycosylated hemoglobin.

First trimester		Second trimester		Third trimester	
Glycosylated hemoglobin (%)	Glycated Albumin (%)	Glycosylated hemoglobin (%)	Glycated Albumin (%)	Glycosylated hemoglobin (%)	Glycated Albumin (%)
6.6 ± 0.9	16.2 ± 2.8	6.2 ± 0.3	16.3 ± 0.9	7.0 ± 0.9	18.4 ± 5.9

### Tissue Paraffin Section Preparation

Placental samples were collected within 0.5 h after cesarean section delivery. Full-thickness placental specimens were obtained from the area near the umbilical cord. Tissue sections of 4 μm thickness were cut from paraffin-embedded placental tissue and mounted onto glass slides.

### Multicolor Immunofluorescence

Consecutive staining was performed by heat-induced antigen retrieval followed by incubation with primary antibody (anti-CD163, 1:200, ab182422, Abcam, Cambridge, MA, USA). The signal was amplified and detected with Opal™ polymer horseradish peroxidase and Opal 570 (1:100, Akoya Biosciences, Japan). The sections were then subjected to heat-induced antibody stripping and incubated with the next antibody (anti-DC-SIGN was used at a dilution of 1:100 and detected with Opal 520; then, anti-CD68 was used at a dilution of 1:200 and detected with Opal 620; and finally, anti-CD14 was used at a dilution of 1:200 and detected with Opal 650) and spectral DAPI (anti-DC-SIGN, ab218419; anti-CD68, ab213363; anti-CD14, ab133503, Abcam, Cambridge, MA, USA). All Opal reagents were used at a dilution of 1:100 (Opal 520, Opal 620 and Opal 650, Akoya Biosciences, Japan). The slides were finally mounted in antifade mounting medium (Akoya Biosciences, Japan) and scanned with a Vectra Polaris (Akoya Biosciences, Japan). The biomarkers to define macrophage populations are summarized in **Table 3**. Meanwhile, the biomarkers of M1 macrophage were recorded as the control. Digital pathology methods were used to determine the density of positive cells. All analyses were performed with Image-pro Plus.

**Table 3:** Surface markers of macrophage to define macrophage populations.

Macrophage type	M1	M2	M2a	M2b	M2c
Surface markers	CD80	CD163	CD163	CD163	CD163
	CD86		DC-SIGN	CD86	CD206

### Immunohistochemistry

Immunohistochemistry of paraffin section (IHC-P) studies were performed on 4-μm-thick tissue sections. The sections were performed by heat-induced antigen retrieval followed by incubation with primary antibody. The sections were incubated with CD80 monoclonal antibody (1/100 dilution, TA501576 ORIGENE, Beijing, China), CD86 monoclonal antibody (1/100 dilution, ab269587 Abcam, Cambridge, MA, USA) and CD206 monoclonal antibody (1/10,000 dilution, 60143-1-Ig Proteintech, Wuhan, China) overnight at 4°C temperature. Second antibody (PV-6002,China) was from the Zhong Shan Golden Bridge Company (Beijing, China). DAB was used as the chromogen. The sections were then counterstained with hematoxylin and mounted with neutral resin size.

### Statistical Analysis

SPSS v18.0 software (Corp.: IBM; Armonk, USA) was used for statistical description and analysis. Continuous variables are represented as the median and range, and categorical or ranked variables are described as the frequency and percentile. The Mann–Whitney U-test or Fisher’s exact test was used to test the differences. All reported P-values are two sided, and P <0.05 was considered statistically significant.

### Data and Resource Availability

The data sets used and/or analyzed during the current study are available from the corresponding author on reasonable request.

## Results

### Distributional Characteristics of M1 and M2 Macrophages in the Chorionic Villi and Decidua

**Figure 1:**
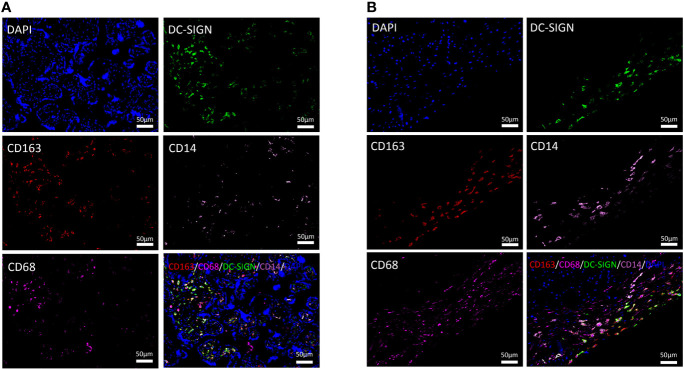
Distributional characteristics of M2 macrophages, M2a macrophages, CD68^+^ macrophages, and CD14^+^ macrophages in the chorionic villi and decidua. **(A)** M2 macrophages (CD163 positive) were mainly infiltrated around vessels and syncytiotrophoblasts. DC-SIGN was located on HBCs in the chorionic villi. CD14 was detected to partially coexpress with CD163 and DC-SIGN in the chorionic villi. The expression degree of CD68 was similar to that of DC-SIGN and CD14 but lower than that of CD163 in the same area of the chorionic villi. **(B)** M2 macrophages (CD163 positive) were prominent in the decidua. Most DC-SIGN-positive cells coexpressed CD163 in the decidua. CD14 was detected to partially coexpress with CD163 and DC-SIGN in the decidua.

We used CD80 and CD86 to localize M1 macrophages. There were only few M1 macrophages distributed in the placenta of both the healthy and uncontrolled T2DM groups (**Figures S1A, B**). The multicolor immunofluorescence was performed in the chorionic villi and decidua to record the general M2 macrophage (CD163) and M2a macrophages (CD163 and DG-SIGN) (**Figure 1**). Immunohistochemistry was performed to record the M2c macrophages (CD206) (**Figure S1C**) and M2b macrophages (CD163 and CD86), which shared the same marker (CD86) with M1 macrophages (22) (**Figure S1B**). In the chorionic villi, the most common infiltrated macrophages were the general M2, which were also responsible for the overall number of HBCs (26). In both of the healthy and uncontrolled T2DM pregnant women, the predominant subtype of M2 macrophages in the chorionic villi (HBCs) was M2a, where it mainly infiltrated around vessels and syncytiotrophoblasts (**Figure 1A**). There were few M2b macrophages (**Figure S1B**) and moderate M2c macrophages distributed in the chorionic villi and decidua of both groups (**Figure S1C**). When it came to the placental decidua, the general M2 macrophages also accounted for the most of infiltrated macrophages (**Figure 1B**).

CD14, a marker of monocytes, was partially expressed on M2a macrophages (CD163 and DC-SIGN double positive) in the chorionic villi and decidua (**Figures 1A, B**). In previous studies, CD68, a general macrophage marker, was expressed with low degree in the placenta (26). In the present study, the distributional of CD68^+^ macrophages were less than M2 macrophages in the same area of the placenta (**Figures 1A, B**).

### The Differences in Distribution of M2 Macrophages, Its Subtypes, CD68^+^ Macrophages, and CD14^+^ Macrophages in the Chorionic Villi of the Placenta in the Normal and Uncontrolled T2DM Groups

**Figure 2:**
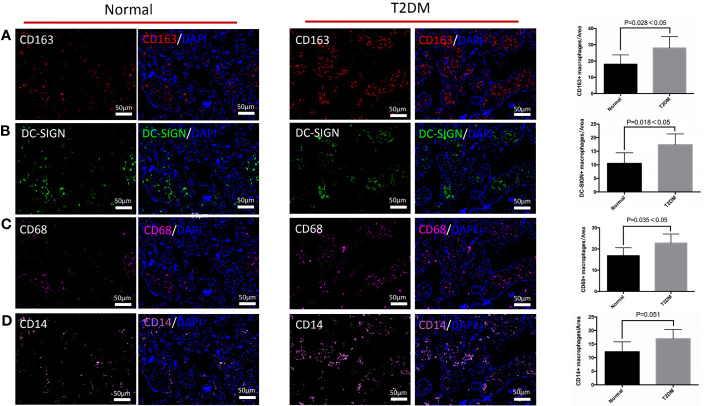
The differences in distribution of M2 macrophages, M2a macrophages, CD68^+^ macrophages, and CD14^+^ macrophages in the chorionic villi of the pregnant women in the normal and uncontrolled T2DM groups. **(A)** The chorionic villi of the uncontrolled T2DM pregnant women presented more M2 macrophages (CD163 positive) than the normal group. **(B)** The chorionic villi of the uncontrolled T2DM pregnant women presented more M2a macrophages (CD163 and DC-SIGN both positive) than the normal group. **(C)** Increased distributional of CD68^+^ macrophages were detected in the chorionic villi of the placenta of the uncontrolled T2DM group. **(D)** The single expression of CD14 was comparable between the two groups.

**Figure 3:**
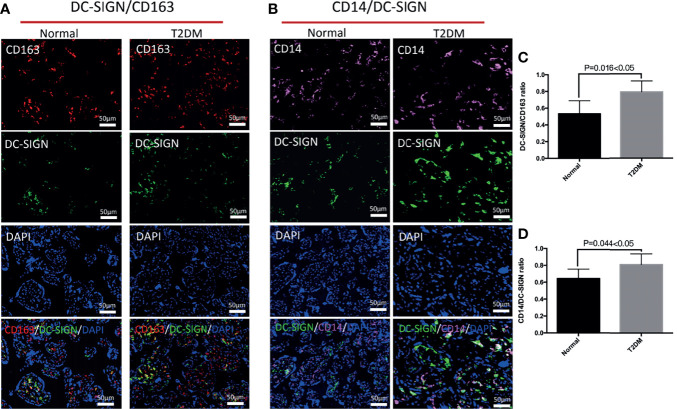
The differences in distribution of M2a macrophages and CD14^+^ M2a macrophages in the chorionic villi of the normal and uncontrolled T2DM placenta. **(A, C)** The number of M2a macrophages was higher in the chorionic villi of the uncontrolled T2DM placenta than the normal placenta. P < 0.05 was considered statistically significant. **(B, D)** The number of CD14^+^ M2a macrophages was significantly higher in the chorionic villi of the uncontrolled T2DM group than the normal group. P < 0.05 was considered statistically significant.

We explored the influences of uncontrolled T2DM on the distributional characteristics of M2 macrophages and its subtypes in the chorionic villi (**Figure 2**). Although the general M2 macrophages, especially the M2a subtype, accounted for the predominant macrophages in healthy and uncontrolled T2DM pregnant women, the uncontrolled T2DM pregnant women had more M2a macrophages infiltration than the healthy pregnant women (p = 0.016) (**Figures 2A, B**). Additionally, the scope of M2a macrophages was also more extensive in the chorionic villi of the placentas of the uncontrolled T2DM group than the normal group (**Figures 3A, C**). There were comparably few M2b macrophages that infiltrated the chorionic villi of both groups (**Figure S1B**). The infiltrated degree of M2c macrophages were moderate and the uncontrolled T2DM pregnant women had similar amounts of M2c macrophage infiltration in the chorionic villi (p = 0.158) (**Figure S1C**).

Furthermore, an increased distribution of CD68^+^ macrophages was detected in the chorionic villi of the placenta of the uncontrolled T2DM group (p = 0.035) (**Figure 2C**). A previous study indicated that HBCs might play a role in the immunology of pregnancy (27). We quantified and compared the CD14^+^ M2a macrophages between the normal and the uncontrolled T2DM groups. Although the HBCs which singly expressed CD14 were comparable between the two groups (p = 0.051) (**Figure 2D**), the number of CD14^+^ M2a macrophages was increased in the uncontrolled T2DM group than in the normal group (p = 0.044) (**Figures 3B, D**).

### The Differences in Distribution of M2 Macrophages, Its Subtypes, CD68^+^ Macrophages, and CD14^+^ Macrophages in the Decidua of the Placenta in the Normal and Uncontrolled T2DM Groups

**Figure 4:**
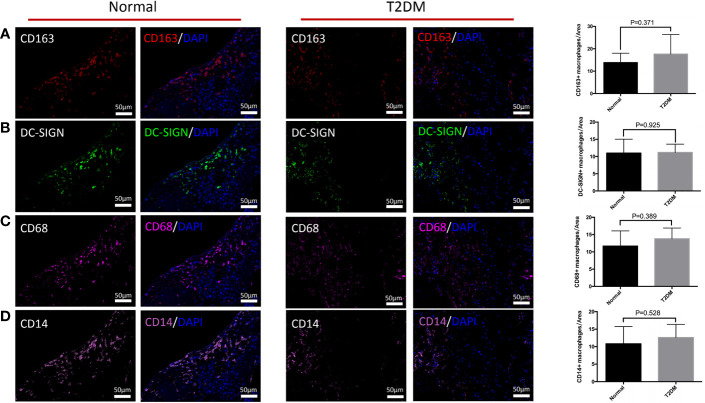
The differences in distribution of M2 macrophages, M2a macrophages, CD68^+^ macrophages, and CD14^+^ macrophages in the decidua of the pregnant women in the normal and uncontrolled T2DM groups. **(A)** The variation of M2 macrophages was not obvious in either the normal or uncontrolled T2DM group. **(B)** The variation of M2a macrophages was not obvious in either the normal or uncontrolled T2DM group. **(C)** No significant differences in the number of CD68^+^ macrophages. **(D)** No significant differences in the number of CD14^+^ macrophages.

**Figure 5:**
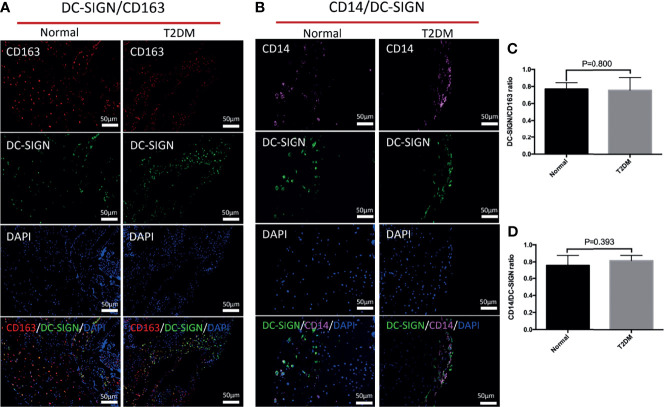
The differences in distribution of M2a macrophages and CD14^+^ M2a macrophages in the decidua of the normal and uncontrolled T2DM placenta. **(A, C)** The M2a macrophages in the decidua of the uncontrolled T2DM group were similar to those of the normal group. P < 0.05 was considered statistically significant. **(B, D)** The CD14^+^ M2a macrophages in the decidua of the uncontrolled T2DM group were similar to those of the normal group. P < 0.05 was considered statistically significant.

We explored the influences of uncontrolled T2DM on the distributional characteristics of M2 macrophages in the decidua (**Figure 4**). The variation of M2 macrophages and M2a macrophages were not obvious in either the normal or uncontrolled T2DM group (**Figures 4A, B**). The M2a macrophages in the decidua of the uncontrolled T2DM group were similar to those of the normal group (p = 0.800) (**Figures 5A, C**). The distributional characteristics of M2b and M2c macrophages in the decidua were in similar cases with that in the chorionic villi. Moreover, no significant differences in the number of CD68^+^ macrophages (p=0.389) (**Figure 4C**), CD14^+^ macrophages (p=0.528) (**Figure 4D**) or CD14^+^ M2a macrophages (p=0.393) (**Figures 5B, D**) were observed between the two groups.

## Discussion

In our study, we examined different phenotypes of macrophages in the chorionic villi and decidua between the placenta of uncontrolled T2DM pregnant women and the normal group. We found that the prominent type of M2 macrophages and their subtype characteristics were altered when hyperglycemia was out of control. In the present study, multicolor immunofluorescence was performed to locate HBCs more precise and to analyze their subtype in uncontrolled T2DM pregnant women and a normal group. We found that most CD163^+^ macrophages were coexpressed with DC-SIGN in the placenta of pregnant women with uncontrolled T2DM. We also found that the numbers of infiltrated general M2 macrophages (CD163 positive) and M2a macrophages (CD163 and DC-SIGN double positive) were both increased in the placental chorionic villi of the women with uncontrolled T2DM compared with the women in the normal group. These results indicated that DC-SIGN^+^ macrophages functioning as M2a macrophages predominated in the placental chorionic villi of women with uncontrolled T2DM. The distributional characteristics of M2 macrophages in the placental chorionic villi and decidua in the term pregnant women with uncontrolled type 2 diabetes mellitus have been revealed for the first time.

The Schliefsteiner et al. study compared the term pregnant women with normal placenta, and found that the placenta of pregnant women with GDM had no significant changes in M2 macrophages but exhibited increased M2a and M2b macrophages in chorionic villi (22). First, in the research of Schliefsteiner et al. (22), the level of blood glucose was controlled in the pregnant women who were diagnosed with GDM. By comparison, the uncontrolled T2DM pregnant women in the present study had progestational existence of T2DM and hyperglycemia was out of control throughout pregnancy. In our research, hyperglycemia in T2DM lasted longer from early pregnancy to late pregnancy and was more severe than GDM. It is a good model to explore the effects of hyperglycemia on the distribution of macrophages in the placenta. Second, in the first trimester of pregnancy, HBCs came about from mesenchymal progenitor cells. In the second and third trimesters, HBCs differentiated from monocytes, which were recruited from the fetal circulation to the placenta (10–12). The effect of uncontrolled T2DM on placental macrophages may develop early in pregnancy. Third, in the present study, we used multicolor immunofluorescence to explore the co-localization of different subtypes of macrophages. It is more accurate in positioning than serial sections. To evaluate CD14^+^ M2a macrophages in the term pregnant women with uncontrolled hyperglycemia throughout pregnancy, we calculated CD14+/DC-SIGN+ macrophages in the placenta of the pregnant women in the uncontrolled T2DM and the normal groups. Interestingly, CD14^+^ M2a macrophages were more highly infiltrated in the placenta of the pregnant women in the uncontrolled T2DM group than in the normal group, although CD14^+^ macrophages were not different between these two groups. CD14, a component of the lipopolysaccharide receptor complex, is an activation marker for macrophages with Toll-like receptor-mediated responses in HBCs (28, 29). Yang et al. found a significant decline in CD14^+^ M2a macrophages in pregnant women with preeclampsia compared with those in the term pregnant women with normal placenta (27). He suggested that CD14^+^ M2a macrophages might play a crucial immunological role in pregnancy (27). Work on human CD14^+^ M2a macrophages has been descriptive, and the function of CD14^+^ M2a macrophages should be examined further.

Obviously, in the chorionic villi, the most common infiltrated macrophages was the general M2, definitely the M2a subtype in both of the groups but not the M1 macrophage, M2b macrophage or M2c macrophage. Compared with the healthy group, uncontrolled T2DM pregnant women had more M2a macrophages where it mainly infiltrated around vessels and syncytiotrophoblasts. Accordingly, two distinct states of macrophage polarization have been defined: M1 macrophages are activated classically and M2 macrophages are activated alternatively depending on their phenotype and functional properties. According to the published literatures, M2 macrophages, which produce molecules and cytokines, play a key role in placental development (19). M2a macrophages possess tissue and vascular remodeling and immunoregulating features; M2b phenotype support humoral immunity and allergic reactions; M2c macrophages induce anti-inflammatory reactions by remodeling of the extracellular matrix and suppression of immunity (19). In the present study, the chorionic villi of placenta in uncontrolled T2DM group tended to have thick vascular wall and more collagen deposition. However, the definite mechanism behind more M2a macrophages infiltration need further exploration.

Of note, the number of M2 macrophages, M2a macrophages, CD14^+^ macrophages and CD68^+^ macrophages in the decidua were not different between the two groups. In contrast, Barke indicated a significantly greater abundance of CD163^+^ macrophages within the decidua of GDM pregnant women (21). In addition to the effects of hyperglycemia on the distribution of macrophages in the decidua, the influence of delivery mode cannot be ignored. Placentae were obtained after both cesarean sections and vaginal deliveries in Barke’s study, but placentae in our study were all obtained from cesarean sections. Vaginal labor and noninfectious preterm labor have been associated with the selective accumulation of decidual macrophages in comparison with elective cesarean section (30).

There are also some different viewpoints about the densities of CD14^+^ macrophages in the decidua. An increased level of CD14 transcription within GDM placental decidua has been reported (31). However, our study explored the expression of CD14 at the protein level in the decidua.

The limitation of this study is the small sample size. Because uncontrolled T2DM pregnant women throughout pregnancy are scarce, we only evaluated 5 pregnant women with complete clinical information and placental specimens, which limited the conclusion of the present study.

## Conclusion

The placenta of uncontrolled T2DM pregnant women exhibit increased M2a macrophages. The subsets of macrophages in the placental decidua are similar between uncontrolled T2DM pregnant women and normal groups. These results may provide a basis for further research on different subsets of macrophages in the placental chorionic villi and decidua.

## Data Availability Statement

The raw data supporting the conclusions of this article will be made available by the authors, without undue reservation.

## Ethics Statement

The studies involving human participants were reviewed and approved by the Biomedical Research Ethics Committee, Peking University First Hospital. The patients/participants provided their written informed consent to participate in this study.

## Author Contributions

MZ contributed to do the experiment and wrote the paper. DC contributed to do the experiment. HY contributed to design the experiment and wrote the paper. All authors listed have made a substantial, direct, and intellectual contribution to the work and approved it for publication.

## Funding

The study was supported by the National Natural Science Foundation of China (81830044).

## Conflict of Interest

The authors declare that the research was conducted in the absence of any commercial or financial relationships that could be construed as a potential conflict of interest.

## Publisher’s Note

All claims expressed in this article are solely those of the authors and do not necessarily represent those of their affiliated organizations, or those of the publisher, the editors and the reviewers. Any product that may be evaluated in this article, or claim that may be made by its manufacturer, is not guaranteed or endorsed by the publisher.
