# Supplementary figures and images for: The Distributional Characteristics of M2 Macrophages in the Placental Chorionic Villi are Altered Among the Term Pregnant Women With Uncontrolled Type 2 Diabetes Mellitus
Source: Front Immunol. 2022 Mar 21;13:837391. doi: 10.3389/fimmu.2022.837391 (PMC8978304; doi:10.3389/fimmu.2022.837391)

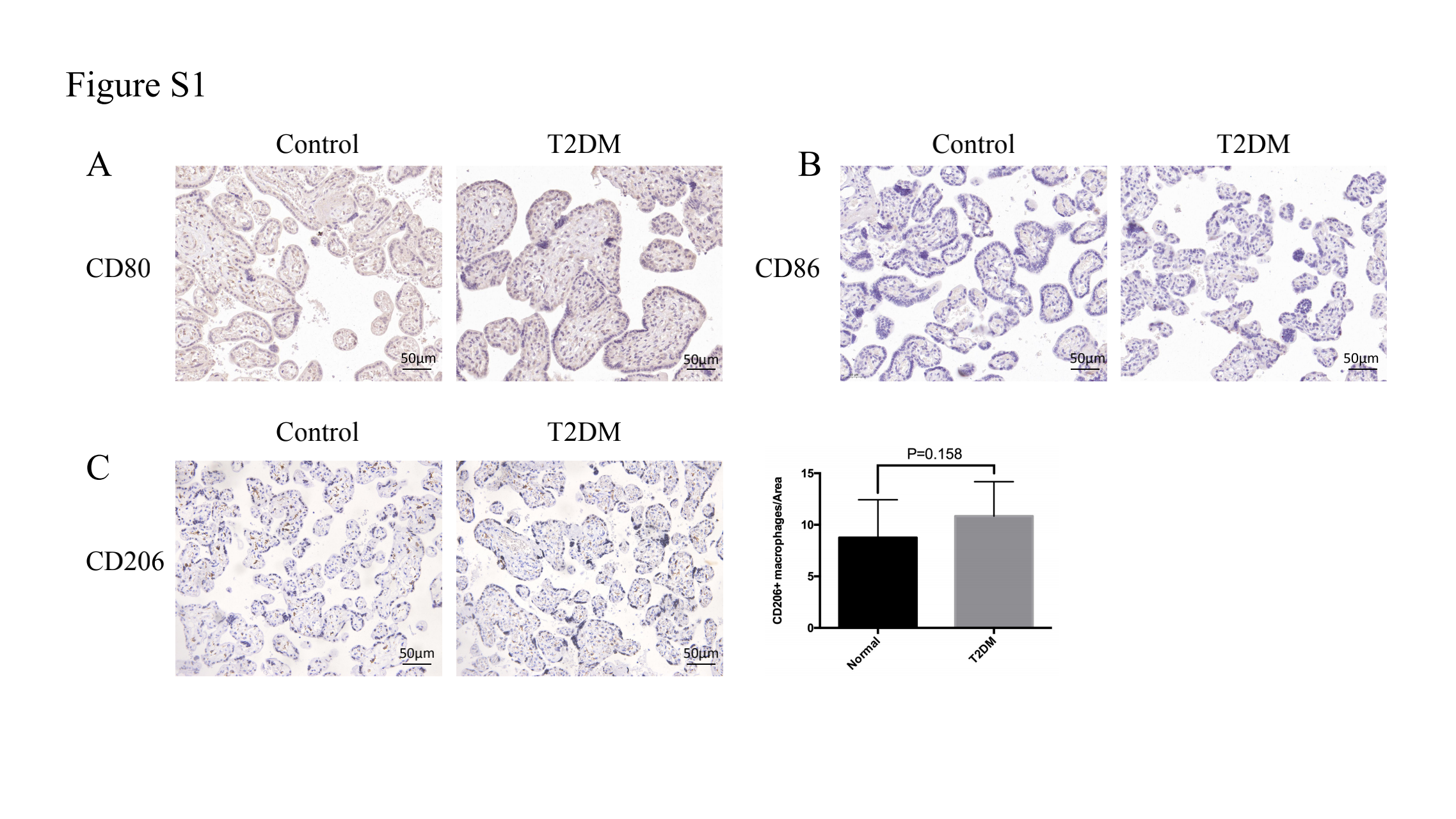

Supplement: Supplementary file 1 [file Image_1.tif]
